# Supplementary material for: Time-Resolved Temperature Mapping Leveraging the Strong Thermo-Optic Effect in Phase-Change Materials
Source: ACS Photonics. 2023 Sep 29;10(10):3576–85. doi: 10.1021/acsphotonics.3c00620 (PMC10588450; doi:10.1021/acsphotonics.3c00620)
Supplement: Supplementary file 2 — ph3c00620_si_002.pdf [file ph3c00620_si_002.pdf]

Time-resolved temperature mapping leveraging the strong thermo-optic effect in phase-change materials

Supplementary Materials

Nicholas A. Nobile[1], John R. Erickson[1], Carlos Ríos[2,3], Yifei Zhang[4], Juejun Hu[4], Steven A. Vitale[5], Feng Xiong[1], Nathan Youngblood[1,*]

[1]Univ. of Pittsburgh, Dept. of Electrical & Computer Engineering, Pittsburgh, PA 15261, USA,
[2]Univ. of Maryland, Dept. of Materials Science & Engineering, College Park, MD, 20742, USA
[3]Univ. of Maryland, Institute for Research in Electronics & Applied Physics, College Park, MD, 20742, USA
[4]MIT, Dept. of Materials Science & Engineering, Cambridge, MA, 02139, USA
[5]Advanced Materials and Microsystems Group, MIT Lincoln Laboratory, Lexington, MA, 02421, USA

*Corresponding email: nathan.youngblood@pitt.edu

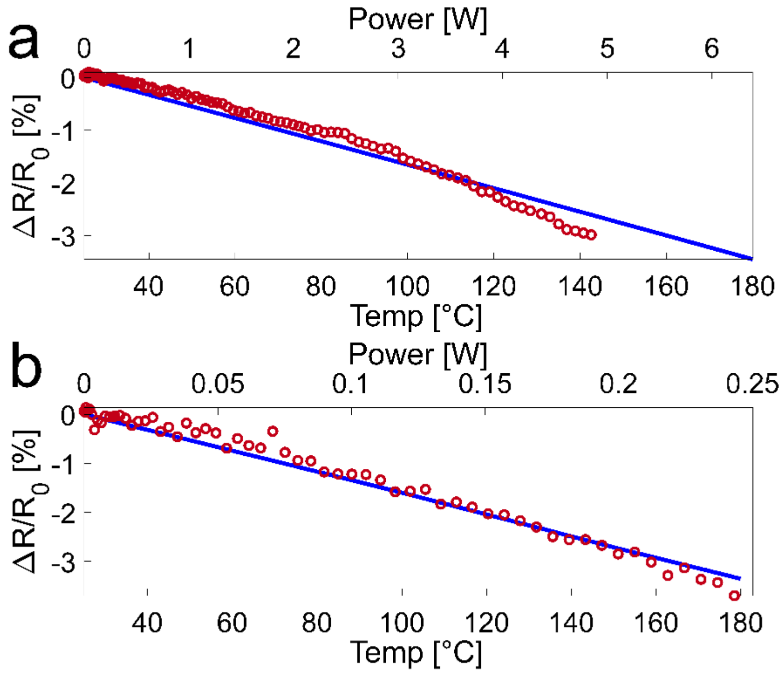

Figure S1: Correlating thermo-optic coefficients to electrical bias. (a-b) Reflectance versus temperature for the platinum heater stack **(a)** and the silicon heater stack **(b)** at 637nm as based on TMM calculations. Values of $n(T_0)$, $k(T_0)$, β, and γ were obtained from ellipsometry measurements and resulted in a highly first-order response which matches expected behavior, especially in the silicon heaters. The Platinum heater design deviates further from the linear model likely due to unintentional substrate heating.

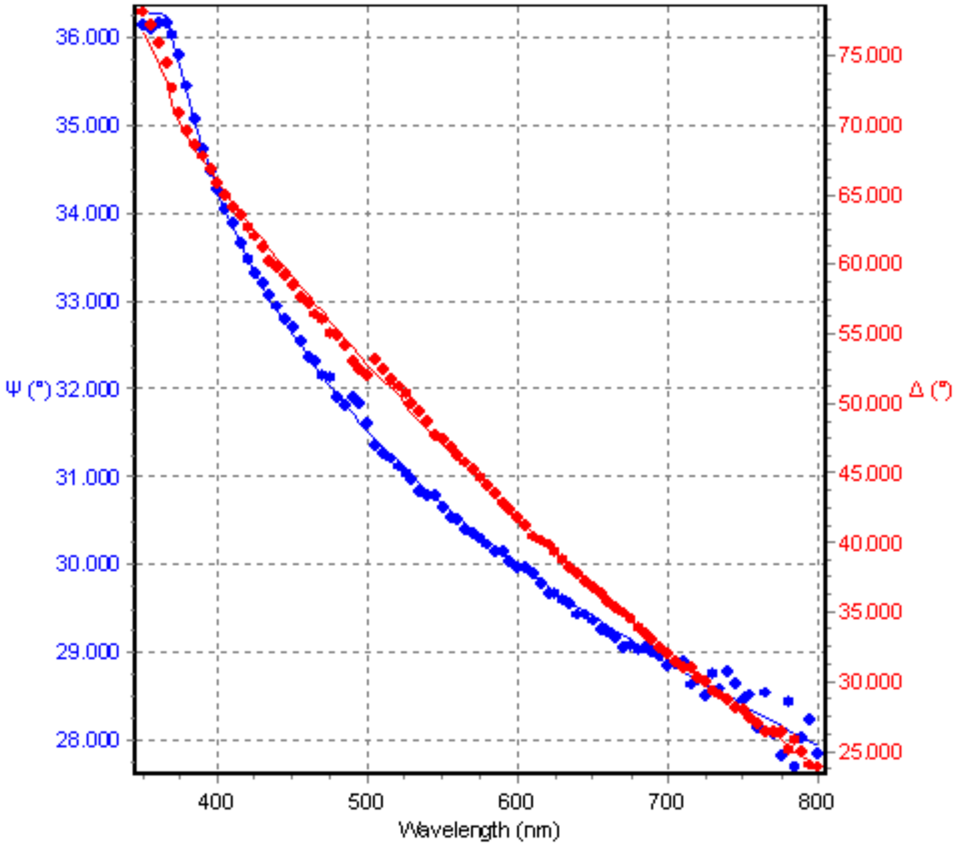

Figure S2: Ellipsometer fitting of optical constants Ψ and Δ at 40.3C.
